# Modulation of Intracellular Quantum Dot to Fluorescent Protein Förster Resonance Energy Transfer via Customized Ligands and Spatial Control of Donor–Acceptor Assembly

**DOI:** 10.3390/s151229810

**Published:** 2015-12-04

**Authors:** Lauren D. Field, Scott A. Walper, Kimihiro Susumu, Eunkeu Oh, Igor L. Medintz, James B. Delehanty

**Affiliations:** 1 Center for Bio/Molecular Science and Engineering, Code 6900, U.S. Naval Research Laboratory, 4555 Overlook Ave, S.W., Washington, DC 20375, USA; lauren.field@nrl.navy.mil (L.D.F.); scott.walper@nrl.navy.mil (S.A.W.); igor.medintz@nrl.navy.mil (I.L.M.); 2 Sotera Defense Solutions, Inc., 7230 Lee DeForest Drive, Columbia, MD 21046, USA; susumu@ccs.nrl.navy.mil; 3 Optical Sciences Division, Code 5600 U.S. Naval Research Laboratory, Washington, DC 20375, USA; eunkeu.oh.ctr.ks@nrl.navy.mil

**Keywords:** fluorescence, sensor, FRET, quantum dot, protein, membrane, assembly, noncovalent, ligand

## Abstract

Understanding how to controllably modulate the efficiency of energy transfer in Förster resonance energy transfer (FRET)-based assemblies is critical to their implementation as sensing modalities. This is particularly true for sensing assemblies that are to be used as the basis for real time intracellular sensing of intracellular processes and events. We use a quantum dot (QD) donor -mCherry acceptor platform that is engineered to self-assemble *in situ* wherein the protein acceptor is expressed via transient transfection and the QD donor is microinjected into the cell. QD-protein assembly is driven by metal-affinity interactions where a terminal polyhistidine tag on the protein binds to the QD surface. Using this system, we show the ability to modulate the efficiency of the donor–acceptor energy transfer process by controllably altering either the ligand coating on the QD surface or the precise location where the QD-protein assembly process occurs. Intracellularly, a short, zwitterionic ligand mediates more efficient FRET relative to longer ligand species that are based on the solubilizing polymer, poly(ethylene glycol). We further show that a greater FRET efficiency is achieved when the QD-protein assembly occurs free in the cytosol compared to when the mCherry acceptor is expressed tethered to the inner leaflet of the plasma membrane. In the latter case, the lower FRET efficiency is likely attributable to a lower expression level of the mCherry acceptor at the membrane combined with steric hindrance. Our work points to some of the design considerations that one must be mindful of when developing FRET-based sensing schemes for use in intracellular sensing.

## 1. Introduction

Increasing the efficacy of theranostic probes, those that can simultaneously sense/report on as well as treat a disease state, is currently a major focus of biomedical research [1,2]. In order to effectively design new and even more efficient theranostic materials, one needs a thorough understanding of the complex intracellular processes that are to be targeted. Historically, fluorescence-based approaches have been used to probe and monitor these processes, which necessitates efficient means of delivering/localizing the fluorophore to the targeted molecule of interest. Various approaches have been employed here and these have ranged from the cellular delivery of fluorophore conjugates directed to epitope tags expressed on targeted proteins to the recombinant expression of the fluorophore tag as a fusion to the protein of interest. Examples of the former include the metal-affinity-driven complexation of nitrilotriacetic acid (NTA)-dye conjugates to targeted proteins bearing cognate polyhistidine sequences [3,4]. Fessenden, for example, used a Cy3-NTA conjugate to probe a dual polyhistidine/green fluorescent protein (GFP)-tagged version of the Ca^2+^ release channel, ryanodine receptor type 1, as a means to probe membrane channel function [5]. Recombinant tagging of targeted proteins for sensing function has also been shown by Yip *et al.* who expressed the channel protein aquaporin as a fusion to the photoconvertible fluorescent protein (mEos2) for the visualization of protein trafficking in response to vasopressin [6]. Other approaches have utilized the *in situ* assembly of the sensing construct. One example is the HaloTag system wherein a modified haloalkane dehalogenase is specifically designed to exogenously bind specified tags that can be linked via a chloroalkane linker to numerous target molecules. Los *et al.* utilized this system to study NF-κB associated cellular processes ranging from DNA-protein complexes to protein translocation [7]. Another example is the FlAsH/ReAsH system developed by Tsien’s group which utilize biarsenical fluorophores that react with vicinal tetracysteine motifs expressed in target proteins [8]. A still further example described by Lee *et al.* used a fluorescent molecular beacon reporter construct comprising a masking/quenching protein, a mitochondrial targeting sequence, a protease-specific cleavage sequence and a GFP reporter [9]. This sensing construct reported on *both* the location and activity level of matrix metalloproteases in living cells.

Despite the demonstrated utility of the aforementioned sensing schemes, they all rely on the use of organic fluorophores or fluorescent proteins which can be limited by their inherent photophysical properties. Chief among these are their susceptibility to photobleaching, potential for chemical degradation and their limited two photon action cross sections which can severely limit deep tissue *in vivo* imaging applications [10]. Luminescent semiconductor nanoscrystals, or quantum dots (QDs), are nanoscale probes whose optical properties are ideal for the real-time, long-term monitoring of cellular processes. These attributes include high quantum yields, large “effective” Stokes shift, and resistance to photobleaching and chemical degradation [11]. Further, their broad absorption that extends into the UV coupled with their narrow, size-tunable emission peaks make them ideal donors for Förster resonance energy transfer (FRET) [12,13]. Finally, their large effective surface area-to-volume ratio makes them an ideal scaffold for the assembly of biologicals around the central QD. To realize their full utility as the basis of intracellular FRET-based sensing (and ultimately in theranostics) one must not only develop a full understanding of how to control the FRET process in the sensing assembly but one must also be able to exert fine control over the intracellular location of the assembled FRET ensemble.

We have previously shown the ability to drive the intracellular assembly of a His_6_-tagged form of the fluorescent protein mCherry to the Ni^2+^-loaded carboxyl termini on the polymer shell of a commercial QD preparation [14]. Using microinjection of QDs coupled with the transient expression of His_6_-mCherry, our results showed the ability to assemble *in situ* a QD-fluorescent protein FRET-based assembly in the cytosol of COS-1 cells. The assembled complexes were stable intracellularly over a 6 h window and it was shown that the QD-sensitized mCherry exhibited an enhanced photostability when excited in a FRET configuration using the QD as donor compared to when the mCherry was excited directly. Despite these positive findings, numerous aspects of the FRET system are worthy of further interrogation in order to fully understand the potential utility of such a system for in real-time intracellular sensing. First, the use of a commercial, polymer-coated QD preparation in that initial experiment did not allow for strict control over the distance between the donor QD surface and the appended mCherry acceptor. Second, control over the intracellular localization of the sensor system was not implemented, with the complexes only being assembled within the cytosol; any effects of intracellular assembly location on the FRET efficiency of the complex were not assessed. Here we build upon those initial studies and continue developing this intracellular assembly approach by performing a detailed examination of the role played by both the QD ligand coating and the assembly location of the QD-mCherry complex on the FRET efficiency of the system. Our working hypothesis in this study is that the nature of the QD ligand coating as well as the location of the FRET assembly can have a profound influence on the resulting FRET efficiency of the system. We make a comparative assessment of the FRET efficiency mediated by a suite of custom ligand molecules when appended to the surface of our in house-synthesized QDs and examine the effects of cytosolic *versus* membrane-directed assembly of the FRET complex. We discuss our results within the context of the considerations that one needs to be mindful of when designing a QD-fluorescent protein FRET ensemble to be used for real-time intracellular sensing.

## 2. Experimental

### 2.1. Materials

Bovine fibronectin, Anotop-10 filters (0.2 µm) and Dulbecco’s phosphate buffered saline (DPBS, 137 mM NaCl, 10 mM phosphate, 3 mM KCl, pH 7.4) were purchased from Life Technologies (Carlsbad, CA, USA). Nalgene syringe filters (0.2 μm) were purchased from Thermo Scientific. The plasmid encoding cytosolic mCherry utilized was previously described [14]. The plasmid encoding mCherry on the cytofacial side of the plasma membrane was generated by cloning the mCherry gene downstream of the extracellular and transmembrane domains of CD1b (CD1b-TMD) [15]. The CD1b-TMD-mCherry-His_6_ construct was synthesized (Genscript Corp., Piscataway, NJ, USA) and cloned into the pcDNA3.1-myc-his(A) vector (Life Technologies) as a *Kpn*I-*Not*I fragment and confirmed by sequencing. This construct resulted in the expression of a C-terminal His_6_ motif to drive QD-mCherry assembly at the membrane. See Supplementary Information Figure S1 for the complete nucleic acid and amino acid sequences. Recombinant mCherry-His_6_ for QD assembly experiments outside cells was expressed and purified as described in reference [14].

### 2.2. QD Synthesis and Capping Ligands

Core-shell CdSe-ZnS QDs were synthesized to have an emission maxima centered at 545 nm and were made hydrophilic by exchange of the native hydrophobic trioctylphosphine/trioctylphosphine oxide (TOP/TOPO) ligands with polyethylene glycol (MW 750; PEG_750_)-appended dihydrolipoic acid (DHLA-PEG_750_-OMe) ligands terminated in methoxy groups as previously described [16]. For comparison, QDs were also capped with either DHLA-PEG_600_ terminated with nitrilotriacetic acid (DHLA-PEG_600_-NTA) or with zwitterionic CL4 capping ligands [17]. In a previous report, DHLA-PEG-NTA ligands were generated “on-QD” using carbodiimide chemistry after the initial cap exchange that removed the native hydrophobic ligands [18]. In this study, the DHLA-PEG_600_-NTA ligand was first synthesized “off-QD” and then used in the cap exchange procedure ( Supplementary Figure S2 for ligand synthesis details). TEM analysis showed that QDs capped with CL4 and DHLA-PEG_600_-NTA had average diameters of 4.6 ± 0.4 nm and QDs capped with DHLA-PEG_750_-OMe had and average diameter of 4.7 ± 0.4 nm (Supplementary Figure S3).

### 2.3. Cell Culture and Transfection

African green monkey kidney (COS-1) cells were cultured in complete growth medium (Dulbecco’s Modified Eagle’s Medium (DMEM; American Type Culture Collection ATCC)) supplemented with 10% (*v*/*v*) heat inactivated fetal bovine serum (ATCC) and 1% (*v*/*v*) antibiotic/antimycotic (Sigma, St. Louis, MO, USA). Cultures were maintained in T25 flasks and incubated at 37 °C under a 5% CO_2_ humidified atmosphere and passaged at 80% confluency. For microinjection, cells were seeded at a density of ~6 × 10^4^ cells/mL per dish onto MatTek™ 14 mm dishes (Ashland, MA, USA), which were coated overnight with fibronectin and allowed to adhere overnight. Transfection of adhered cells was performed by removing the complete growth media and washing the cell monolayers once with serum free DMEM. During this time, plasmid DNA was complexed with Lipofectamine2000™ in serum free DMEM. For each dish, 0.4 μg of DNA was added to 25 μL of serum free DMEM and allowed to equilibrate while 1 µL of Lipofectamine2000™ was mixed carefully with 25 μL of DMEM. After five minutes, the two mixtures were combined and allowed to form DNA-lipid complexes for 20 min at room temperature whereupon 50 μL was added to each well. After a 6 h incubation of the Lipofectamine2000™-DNA complexes with cell monolayers, the transfection media was removed and replaced with complete media and the cells were cultured overnight to allow for mCherry expression.

### 2.4. Microinjection

After successful transfection, cells were injected using an Eppendorf FemtoJet^®^ Microinjector controlled by an InjectMan^®^ NI 2 micromanipulator in a manner similar to that described previously [19]. Cells were washed once with DPBS to remove excess media and injection was performed on cells incubated in filter-sterilized DPBS. The microinjection tip (Eppendorf Femtotips, Hauppauge, NY, USA) was loaded with 10 μL of QDs (0.2 μm filtered). QDs capped with DHLA-PEG_750_-OMe and CL4 were filtered using DPBS alone while QDs capped with DHLA-PEG_600_-NTA were complexed with ~20 equivalents of NiCl_2_ for 20 min and then filtered with 0.5% FBS in DPBS to increase intracellular stability of the QDs upon injection. Injection time and pressure was optimized for each tip loading to ensure cellular viability upon injection and uniform distribution of QDs throughout the cytosol. Typical injection conditions used were 0.6 s at pressures of 600–700 hPa.

### 2.5. Microscopy and Image Analysis

Differential interference contrast (DIC) and epifluorescence microscopy were utilized to determine the QD distribution throughout the cells and visualize the FRET interaction between the injected QDs and the expressed mCherry protein. Images were acquired with an Olympus IX-71 total internal reflection fluorescence microscope equipped with a 60× oil immersion lens. Samples were excited using a metal halide arc lamp and images were collected using the excitation and emission settings described in the Supplementary Information Table S1. Various fields were examined for mCherry expression and injection was performed such that fields of view were comprised of cells containing (1) QD donor only; (2) mCherry acceptor only; and (3) both QD donor and mCherry acceptor. This approach provided the necessary controls for FRET-based image analysis. After injection, fields were imaged for the donor excitation and emission (410 nm and 535 nm, respectively), acceptor excitation and emission (576 nm and 620 nm, respectively) and donor excitation with acceptor emission (410 nm and 620 nm, respectively) in addition to DIC images. Relative quantification of relative acceptor sensitization across ligand species was performed by first performing a background correction of all images followed by normalization of the intensity of the FRET channel to the ligand that mediated the most efficient FRET (CL4 in all cases). All data analyses were performed using Image J (ver. 1.48 v). The images were false-colored and prepared for publication using MetaMorph Advanced software (ver. 7.8.0.0).

### 2.6. Gel Electrophoresis

Gel electrophoresis (1% low EEO agarose) was performed on DHLA-PEG_600_-NTA and CL4 QDs assembled with mCherry protein to demonstrate ratiommetric assembly. QDs (7.5 pmol) were complexed with increasing ratios of mCherry protein in DPBS at QD-mCherry ratios ranging from 1:1 to 1:30 and allowed to incubate for 30 min. Additionally, an mCherry only control was included at the 1:30 mCherry equivalent. QD-protein mixtures were subjected to electrophoresis for 30 min and images were taken at 5 min intervals.

### 2.7. FRET Analysis

To determine the FRET efficiency of the QD-mCherry complexes, QDs were assembled with mCherry protein at QD-mCherry ratios from 1:1 to 1:30 and were allowed to incubate for 30 min. Control solutions containing mCherry alone were included at all mCherry equivalents to determine the contribution of direct excitation of the acceptor in absence of the donor. The assemblies were excited at 400 nm using a Tecan Infinite M1000 microplate reader and emission spectra was collected from 450–700 nm. The FRET efficiency at each QD-mCherry ratio was determined using the equation:

(1) $${\text{FRET}}_{E}=1-\frac{{\text{F}}_{DA}}{{\text{F}}_{D}}$$

where F*_DA_* and F*_D_* are the fluorescence intensity of the QD donor in the presence and absence of the mCherry acceptor, respectively. The QD-mCherry separation (*r*) distance for each ratio was determined according to the equation:

(2) $$E_{n}=\;\frac{n{\left(\frac{R_{0}}{r_{DA}}\right)}^{6}}{1+n{\left(\frac{R_{0}}{r_{DA}}\right)}^{6}}$$

where *R*_0_ is the calculated Förster distance for the donor–acceptor pair. Data analysis was performed in Excel (ver. 14.0).

## 3. Results

### 3.1. Experimental Rational and Design

The design and implementation of a QD-protein FRET ensemble as an effective intracellular sensing modality first requires the careful consideration of two critical elements: (1) the FRET efficiency of the assembly wherein the donor–acceptor distance can be manipulated and (2) control over the intracellular localization of the sensing assembly. Our goal in this study was to assess the ability to control the intracellular FRET efficiency of a QD-mCherry donor–acceptor assembly by modulating the ligand coating on the QD donor while simultaneously site-specifically expressing the mCherry acceptor to control the location of the *in situ* assembled QD-mCherry complex (cytosol *versus* the cytofacial leaflet of the plasma membrane). We began these studies by first performing QD-mCherry assembly and FRET efficiency experiments outside the cellular environment. We subsequently employed microinjection of the QD donor coupled with transient transfection/expression of the mCherry acceptor to determine the efficiency of site-specific intracellular FRET.

**Figure 1:**
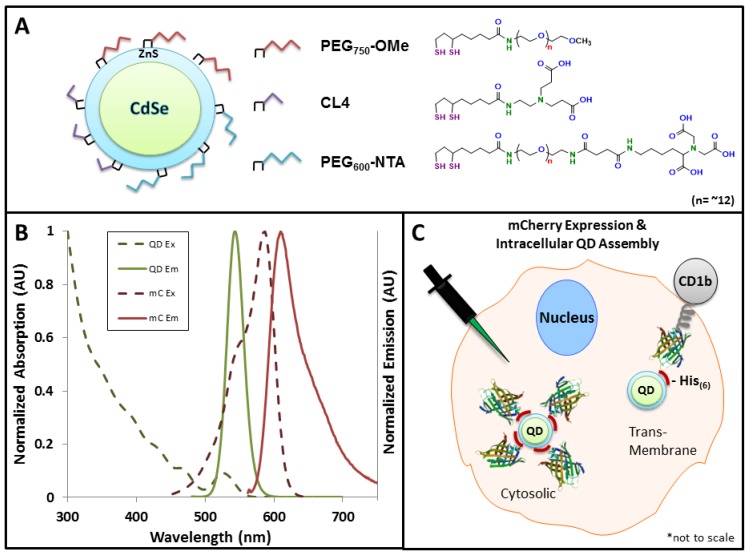
Fluorescent materials and Förster resonance energy transfer (FRET) rationale used in this study. (**A**) Schematic of CdSe-ZnS core-shell quantum dots (QDs) and various capping ligands; (**B**) Absorption and emission spectra of the QD and mCherry (mC) donor–acceptor pair showing significant overlap of the QD emission and mCherry absorption, allowing for FRET-sensitized emission of mCherry; (**C**) Intracellular QD-mCherry assembly strategies. mCherry was expressed either free in the cytosol or as a fusion to the C-terminus of the transmembrane receptor, CD1b. Upon microinjection of QDs, a His_6_ motif on the C-terminus of the mCherry drove the intracellular assembly of mCherry to the QD surface either in the cytosol or at the cytofacial leaflet of the plasma membrane.

The ligands used in this study included: (1) CL4, a small zwitterionic molecule bearing two carboxyl groups and one each secondary and tertiary amine moieties (length = ~1.8 nm) [17]; (2) DHLA-PEG_750_-OME (PEG_750_-OMe; length = ~6.8 nm) [17], a PEGylated ligand terminating with a neutral methoxy group; and (3) DHLA-PEG_600_-NTA (PEG_600_-NTA; length = ~6 nm) [18], a shorter PEGylated ligand that terminates with a negatively charged nitrilotriacetic acid (NTA) function (Figure 1A). All three ligands share the same DHLA anchor wherein the thiol pair mediates stable binding of the ligand to the ZnS shell. This panel of ligands was selected specifically because they have been shown to mediate intracellular QD stability [17] and they span a range of lengths for controlling the donor–acceptor distance in the FRET assembly. It is worth noting that the CL4 and DHLA-PEG750-OME ligands allow for His-based assembly of the mCherry to the ZnS shell (albeit with differing degrees of accessibility of the His_6_ tag to the Zn surface given the varied lengths of the two ligand species). In contrast, the PEG_600_-NTA ligand promotes assembly to the carboxyl groups on the terminal nitrilotriacetic acid moiety. As shown in Figure 1B, FRET in this system is driven by the significant degree of spectral overlap between the emission of the 545 nm QD donor and the absorbance of the mCherry acceptor. Two further features drive efficient FRET in this QD-protein system: (1) the excitation of the QDs in the UV (~350 nm) resulting in negligible direct excitation of the fluorescent protein acceptor and (2) the ability to array multiple mCherry protein acceptors around the central QD scaffold which enhances overall FRET efficiency [20]. Finally, the site-specific assembly of the QD-mCherry FRET ensemble was controlled by expressing the mCherry acceptor in either of two forms; free in the cytosol or appended to the inner leaflet of the plasma membrane via its expression as a fusion to the extracellular and transmembrane domains of CD1b (see Supplementary Information Figure S1). Upon injection of the QDs into the cytosol, the free C-terminal His_6_ domain on either mCherry variant mediated the localized assembly process (Figure 1C).

### 3.2. Efficacy of the FRET System

**Figure 2:**
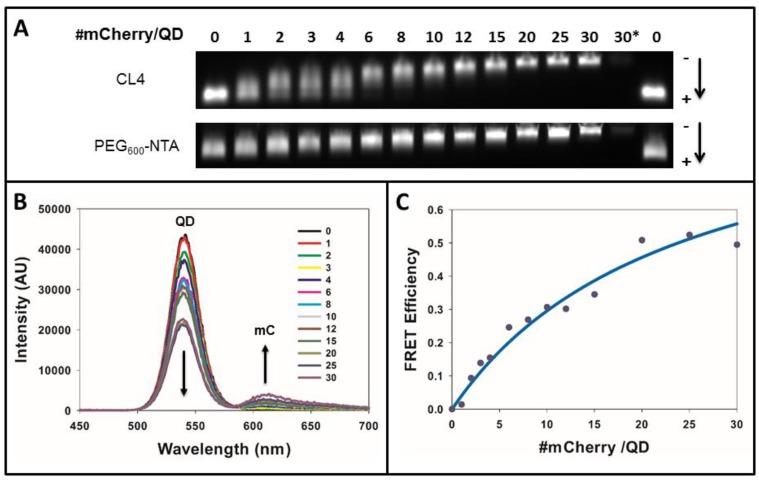
Assembly and FRET analysis of the QD-mCherry (mC) donor–acceptor pair. (**A**) The 545 nm-emitting QDs capped with CL4 or PEG_600_-NTA were assembled with increasing ratios of mCherry and separated on 1% agarose gels; (**B**) Emission spectra of CL4 capped 550 nm QDs showing sensitization of QD donor emission with increasing ratio of mCherry acceptor. Data have been corrected for direct excitation of mCherry; (**C**) Plot of QD-mCherry FRET efficiency as a function of increasing QD-mCherry ratio. Line is fit to Equation (2).

We first performed *in vitro* assembly experiments to ensure that the His_6_-tagged mCherry could successfully assemble to the QD surface. Here, increasing ratios of mCherry were complexed with the CL4- and PEG_600_-NTA-capped QDs and the complexes were subjected to gel electrophoresis. As shown in Figure 2A, QDs bearing either ligand species showed a decreased migration within the gel as increasing numbers of mCherry were added, demonstrating an increase in the size of the QD-mCherry complexes. Analysis of the PEG_750_-OMe QDs was not performed here as they have no net charge and would not migrate when subjected to electrophoresis. Interestingly, assembly of mCherry to the PEG_600_-NTA QDs resulted in discrete gel bands at nearly all QD-mCherry ratios while assembly of the protein to CL4-capped QDs yielded a “smearing” banding pattern at ratios below ~10 indicative of a mixed population of QDs wherein the number of proteins per QD, or valence, varied. This is consistent with a Poisson distribution of assemblies at lower QD-protein ratios [21]. At valences greater than ~10, however, we observed tight, discrete fluorescent bands for QDs capped with both ligands demonstrating the formation of more homogeneous populations of QD-mCherry complexes. These results are not unexpected given the fact that the manner in which the His_6_-tagged mCherry assembles to the QD surface differs slightly between these two QD species. For the PEG_600_-NTA QDs, the NTA groups on the termini of the PEG_600_-NTA are more readily accessible to the His_6_ tag on the mCherry while for the CL4-capped QDs, the binding of the His_6_ tag to the Zn-rich surface of the QD shell requires the interdigitation of the tag through the ligand layer to reach the QD shell. Despite this slight difference, our results clearly show that at mCherry valences >10, similar binding/assembly efficiencies are obtained for both QDs.

Having demonstrated the controlled assembly of QD-mCherry complexes, we next performed FRET analysis to determine the efficiency of the energy transfer process between the QD donor and the mCherry acceptor. For this analysis we opted to use QDs capped with CL4, the shortest ligand used in these studies and, therefore, the species expected to mediate the most efficient FRET. To determine the FRET efficiency (FRET*_E_*) of the QD-mCherry FRET pair, increasing ratios of mCherry were assembled onto the QD surface and the emission spectra of the complexes were read when excited at 400 nm. As increasing numbers of mCherry were arrayed around the central QD, a concomitant decrease in QD emission coupled with a corresponding increase in mCherry emission was observed (Figure 2B). As shown in Figure 2C, a plot of the FRET efficiency (see Experimental) *versus* the QD-mCherry ratio showed that a maximum FRET efficiency of ~50% was achieved when ~20 mCherry proteins per QD were assembled onto the QD surface. This corresponded to a calculated center-to-center QD-mCherry distance of ~8.1 nm (using an *R*_0_ value of 6.5 nm for the FRET pair). This calculated distance agrees well with the known sizes of both the QDs (~3 nm radius) and of mCherry (~3 nm radius from barrel end to protein center) when one factors in the CL4 ligand spacing and the enhanced FRET efficiency driven by the arraying of multiple mCherry acceptors around the QD core. Further, these results agree well with our previous characterization of the packing of mCherry to the surface of QDs of this size [13,22]. We determined the FRET*_E_* for the PEG_600_-NTA QDs and PEG_750_-OMe QDs to be 61% and 38%, respectively (see Supplementary Information Figure S4). We chose to perform microinjection/intracellular assembly experiments using the CL4-capped QDs given the combination of ease of injection, intracellular colloidal stability and FRET*_E_* afforded by these QDs.

### 3.3. Intracellular QD-mCherry Assembly and FRET Efficiency

Having confirmed the successful controlled assembly of His_6_-tagged mCherry to the QD donor surface and determined the FRET efficiency of the resultant ensembles, we next sought to characterize the intracellular FRET efficiency as a function of both the QD ligand coating and the location of the donor–acceptor complex. To achieve this, COS-1 cells were transiently transfected to express His_6_-mCherry either free in the cytosol or appended to the inner leaflet of the plasma membrane as a fusion to the cell surface receptor CD1b. During these studies, we noted that the transfection efficiency across experiments was typically ~60%–70%. Cells were then injected with QDs capped with CL4, PEG_600_-NTA or PEG_750_-OMe ligands. We employed an injection strategy that ensured all the necessary controls were present in a single field of view. Specifically, the injection was executed such that each field of view contained cells with QD donor only (no mCherry acceptor), mCherry acceptor only (no QD donor) or both the QD donor and mCherry acceptor. We then imaged these fields for the direct excitation/emission of the donor and acceptor and the FRET between the QD and mCherry. In this manner, the contribution of the QD donor emission and direct mCherry acceptor emission in the FRET channel could be accounted for in the ensuing image analysis.

**Figure 3:**
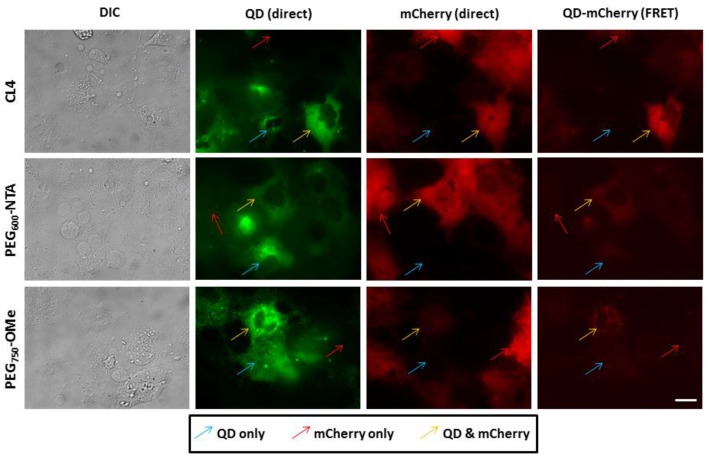
Cytosolic assembly of QDs and mCherry. COS-1 cells expressing His_6_-terminated cytosolic mCherry proteins were microinjected with QDs coated with CL4, PEG_600_-NTA and PEG_750_-OMe. Fields were specifically imaged where cells contained mCherry only (red arrow), QDs only (blue arrow) or both QD and mCherry (yellow arrow) to allow for the appropriate FRET imaging controls. Note the level of efficient FRET present in cells injected with the CL4 QDs, with slightly less efficient FRET observed in cells injected with PEG_600_-NTA QDs. PEG_750_-OMe QDs appeared to mediate the least efficient FRET with intracellular mCherry. Scale bar is 20 µm.

As shown in Figure 3, both the injected QDs and the cytosolically expressed mCherry displayed a diffuse fluorescence pattern with each fluorophore being well distributed throughout the entire cell volume. Interestingly, quantitative analysis of the FRET channel showed clear differences in the ligand-specific FRET efficiency. Specifically, the cells injected with the CL4-coated QDs displayed the most efficient FRET as evidenced by the highest fluorescence intensity in the FRET channel for this ligand compared to the PEG_600_-NTA and PEG_750_-OMe. Relative quantification of the fluorescence intensities in the FRET channel revealed that for the CL4 ligand the QD-mCherry FRET sensitization was ~7-fold greater than PEG_600_-NTA and ~5-fold greater than PEG_750_-OMe (see Supplementary Information Figure S5). Clearly the shorter, compact CL4 ligand (~1.8 nm in length) afforded the more robust assembly of the His_6_-mCherry protein to the QD surface relative to the other two longer ligands whose lengths are greater than 6 nm.

**Figure 4:**
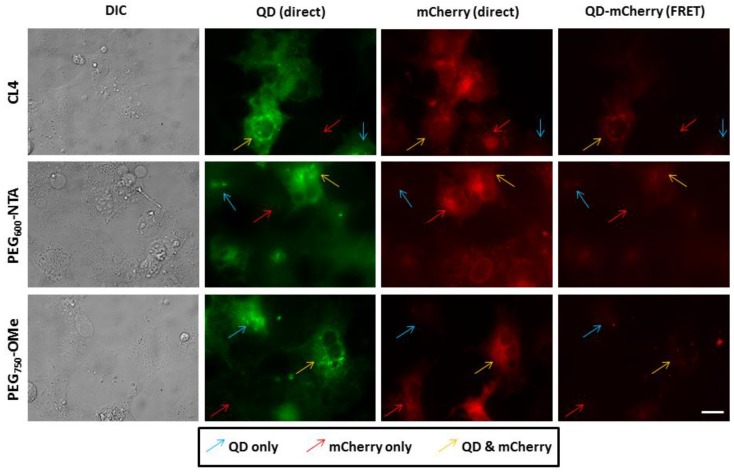
Assembly of QDs and mCherry at the cytofacial leaflet of the plasma membrane. COS-1 cells expressing mCherry proteins on the cytofacial leaflet of the plasma membrane (as a fusion to CD1b) were microinjected with QDs coated with CL4, PEG_600_-NTA and PEG_750_-OMe. Imaging was performed as described for Figure 3. Note the lower level of mCherry expression due to its localization at the plasma membrane. The FRET signal for the CL4 QDs bound to membrane expressed mCherry was ~50% that observed for cytosolic mCherry. Scale bar is 20 μm.

We then expressed mCherry on the inner leaflet of the plasma membrane as a fusion to CD1b and confirmed its localization at the membrane by immunofluorescence analysis (Supplementary Information Figure S6). Here, slightly different FRET results were obtained when QDs bearing the various ligands under study were injected (Figure 4). First, it was apparent that the overall fluorescence intensity of the mCherry (under direct excitation) was reduced 5-fold compared to when it was expressed freely in the cytosol (see Supplementary Information Figure S5). We attributed this lower direct fluorescence emission from the mCherry to the fact that the protein was expressed within the secretory pathway and processed/directed to the membrane which resulted in an overall lower level of intracellular mCherry. It was also clear that the efficiency of QD-mCherry FRET was diminished as a result of the mCherry being expressed appended the inner leaflet of the membrane. Upon direct comparison, the FRET for CL4-capped QDs with membrane-bound mCherry was ~60% that observed for this same donor–acceptor pair in the cytosol (see Supplementary Information Figure S5). Further, the FRET for the PEG_600_-NTA QDs was also markedly reduced and there was no measurable FRET signal at the plasma membrane for QDs capped with PEG_750_-OMe. While the lower level of mCherry expression likely contributed to this, it is also likely that steric hindrance of the mCherry tethered to the inner leaflet played a role in diminishing the ability of the injected QDs to dock onto the terminal His_6_ tag. In this instance, it was only the small CL4 ligand-capped QDs that were able to engage in efficient FRET with membrane-bound mCherry. Taken together with the cytosolic mCherry results, these findings clearly demonstrate that not only can the localized expression of the mCherry acceptor mediate the controlled site-specific assembly of QD-fluorescent protein but also the nature of the capping ligand plays a critical role in the efficiency of FRET once the resulting donor–acceptor complex is formed.

## 4. Discussion

Our need to understand how to exert fine control over nanomaterials when interfaced with cells continues to grow at an almost unabated pace [23,24]. New nanoparticle formulations and delivery modalities necessitate that one have a detailed working knowledge of how to control the delivery, assembly and intracellular fate of nanoparticle–biological ensembles. This is indeed true in the case of nanoparticle-based sensing platforms, particularly those that employ FRET as the readout modality. Multiple factors need to be taken into account including: (1) the facile generation of the FRET donor–acceptor assembly; (2) the delivery of the ensemble sensor; and (3) the efficacy of the sensor *in vitro*. Our goal in this study was to perform a detailed characterization of the efficiency of the intracellular assembly of a QD-fluorescent protein FRET pair. Specifically, we were interested in the role played by the capping ligand on the QD surface in modulating the FRET efficiency in the assembly as well as the ability to site-specifically control the intracellular generation of the FRET complex.

In previous work, we showed the ability to use metal affinity coordination to drive the intracellular *in situ* assembly of microinjected commercial polymer-coated QDs with His_6_-tagged mCherry protein transiently expressed in the cytosol [14]. Despite the positive results obtained in that work, much more remains to be understood in how to control such assemblies intracellularly if they are to be used as the foundation of FRET-based sensing. For example, the proprietary nature of the polymer capping ligand in our previous work did not allow for discrete control of the distance between the QD donor and protein acceptor. Additionally, no strategy was employed to direct the assembly of the FRET complex to a particular subcellular location. Building on the initial success of those studies, our goal here was to perform a more detailed, controlled analysis of the role played by both the QD donor material itself as well as the cellular location of QD-protein assembly. Using the same transient expression/injection approach we first systematically tested a suite of QD-capping ligands for their ability to assemble to and engage in FRET with mCherry proteins outside of the cellular environment. Both a short zwitterionic capping ligand (CL4) and a longer PEGylated ligand showed the ability to mediate assembly of His_6_-tagged mCherry proteins in a quantitative manner. The CL4 ligand facilitated a maximal FRET efficiency of ~50% when ~20 mCherry proteins were arrayed around the central QD. When tested intracellularly, this same CL4 ligand mediated the most efficient rate of FRET of the ligands tested, a testament to the facile and stable assembly of the His_6_ tag to the Zn-rich shell on the QD donor. Interestingly, we noted distinct differences in the FRET efficiency depending on whether the mCherry acceptor was expressed in the cytosol or appended to the inner leaflet of the plasma membrane.

A few salient features of this system bear pointing out. First, the intracellular synthesis of the acceptor portion of the FRET ensemble is at the same time elegant and practical; it obviates the need for assembly of the preformed FRET ensemble outside the cell followed by its subsequent cellular delivery and targeting. Second, the intracellular location of the assembled complex was controlled entirely by manipulating the location of the His_6_-bearing protein acceptor. The success of this approach opens the exciting possibility of other means of directing the site-specific, acceptor-driven QD-protein assembly process. For example, instead of expression as a fusion with a transmembrane receptor, the inclusion of a post-translational modification signal (e.g., palmitoylation [25] or farnesylation [26] signal sequence) or a nuclear localization signal [27] could have been encoded in the expressed mCherry acceptor. Further, the tractable nature of the genetically-encoded mCherry acceptor allows for the modification of the spacer sequence between the His_6_ tag and the mCherry protein. Here, the inclusion of recognition sequences for kinases, proteases or other enzymes can realize the sensing modalities for a variety of cellular processes. It is fundamental studies such as those performed herein that are a critical first step in building the appropriate “toolbox” that will ultimately drive the successful implementation of these QD-based FRET sensors that utilize intracellularly expressed fluorescent proteins as energy acceptors for the sensing of myriad physiological processes.

## 5. Conclusions

In conclusion, here we have examined the intracellular FRET efficiency of an in situ-assembled QD donor-mCherry protein acceptor complex. The mCherry acceptor was transiently expressed either in the cytosol or appended to the inner leaflet of the plasma membrane. Microinjected QDs, each bearing a different surface capping ligand, self-assembled to the mCherry via a terminal polyhistidine epitope on the mCherry acceptor. We determined that intracellular QD-mCherry complexation and the resulting efficiency of energy transfer was most effectively mediated by QDs capped with the small zwitterionic ligand, CL4. We determined that the FRET process occurred more efficiently in the cytosol than at the plasma membrane and this was attributed to a higher level of cytosolically-expressed mCherry protein coupled with increased steric hindrance of the assembly process at the membrane. Cumulatively, our results show the utility of such self-assembled constructs and point to the feasibility of their implementation as intracellular FRET-based sensing platforms.

## Supplementary Files

Supplementary File 1
