# Supplementary material for: Modulation of Intracellular Quantum Dot to Fluorescent Protein Förster Resonance Energy Transfer via Customized Ligands and Spatial Control of Donor–Acceptor Assembly
Source: Sensors (Basel). 2015 Dec 4;15(12):30457–68. doi: 10.3390/s151229810 (PMC4721730; doi:10.3390/s151229810)
Supplement: Supplementary File 1 [file sensors-15-29810-s001.pdf]

Supplementary Information: Modulation of Intracellular Quantum Dot to Fluorescent Protein Förster Resonance Energy Transfer via Customized Ligands and Spatial Control of Donor-Acceptor Assembly

Lauren D. Field, Scott A. Walper, Kimihiro Susumu, Eunkeu Oh, Igor L. Medintz and James B. Delehanty

1. Supporting Information Materials and Methods

Chemicals. *N,N′*-dicyclohexylcarbodiimide (DCC) and 4-(dimethylamino)pyridine (DMAP) were purchased from Acros Organics (Fisher Scientific, Pittsburgh, PA, USA). Tris(2-carboxyethyl) phosphine hydrochloride (TCEP·HCl) was purchased from Carbocore (The Woodlands, TX, USA). H_2N-NTA(OMe)(OEt)$_2$ and TA-PEG$_{600}$-COOH were synthesized as previously reported [1–3]. All the other chemicals including solvents were purchased from Sigma Aldrich (St. Louis, MO, USA) or Acros Organics and used as received.

Instrumentation. ^{1}H NMR spectra were recorded on a Bruker SpectroSpin 400 MHz spectrometer. Chemical shifts for ^{1}H NMR spectra are reported relative to tetramethylsilane (TMS) signal in deuterated solvent (TMS, δ = 0.00 ppm). All *J* values are reported in hertz. Finnigan LCQ Classic electrospray ionization/ion trap mass spectrometer was used for mass spectral analysis. Each sample was dissolved in methanol, and introduced by direct infusion using a syringe pump. Electronic absorption spectra were recorded using an HP 8453 diode array spectrophotometer (Agilent Technologies, Santa Clara, CA, USA) or Shimadzu UV-1800 UV-vis spectrophotometer. Fluorescence spectra were collected using a Spex Fluorolog-3 spectrophotometer (Jobin Yvon Inc., Edison, NJ, USA) equipped with a red-sensitive R2658 Hamamatsu PMT detector.

Ligand Synthesis. DHLA-PEG$_{600}$-NTA(OMe)(OEt)$_2$ was synthesized as described below. The final form (DHLA-PEG$_{600}$-NTA) was prepared via ester hydrolysis after the QDs were ligand exchanged with DHLA-PEG$_{600}$-NTA(OMe)(OEt)$_2$.

TA-PEG$_{600}$-NTA(OMe)(OEt)$_2$. H_2N-NTA(OMe)(OEt)$_2$ (0.203 g, 6.11×10^{-4} mol), DCC (0.151 g, 7.32×10^{-4} mol), DMAP (18.3 mg, 1.50×10^{-4} mol) and CH_2Cl_2 (10 mL) were added to a 100-mL round-bottom flask equipped with an addition funnel, and the reaction vessel was purged with N_2. TA-PEG$_{600}$-COOH (0.506 mL, ~5.77×10^{-4} mol) dissolved in 10 mL of CH_2Cl_2 was added dropwise at room temperature. After 9 h, H_2N-NTA(OMe)(OEt)$_2$ (53.6 mg, 1.61×10^{-4} mol) and DCC (32.4 mg, 1.57×10^{-4} mol) dissolved in 5.0 mL of CH_2Cl_2 was further added dropwise. The reaction mixture was stirred overnight under N_2. The white precipitate was filtered off and the solvent was evaporated. The residue was chromatographed on silica gel with $CHCl_3$:MeOH (10:1). Yield = 0.477 g (~69% based on 0.506 g of TA-PEG600-COOH). ^{1}H NMR (400 MHz, $CDCl_3$): δ 6.65 (br s, 1H, N*H*), 6.45 (br s, 1H, N*H*), 6.29 (br s, 1H, N*H*), 4.14 (q, 4H, *J* = 9.6 Hz, –OC*H*$_2$CH$_3$), 3.6–3.75 (m), 3.51–3.59 (m, 5H), 3.37–3.51 (m, 5H), 3.15–3.3 (m, 2H), 3.07–3.22 (m, 2H), 2.52 (s, 4H), 2.41–2.50 (m, 1H), 2.20 (t, 2H, *J* = 9.8 Hz, –C*H*$_2$CO–), 1.85–1.95 (m, 1H), 1.60–1.77 (m, 6H), 1.38–1.58 (m, 6H), 1.26 (t, 6H, *J* = 9.6 Hz, –OCH$_2$C*H*$_3$).

DHLA-PEG$_{600}$-NTA(OMe)(OEt)$_2$. TA-PEG600-NTA(OMe)(OEt)$_2$ (0.247 g, ~2.07×10^{-4} mol), ethanol (2.5 mL) and deionized water (1.0 mL) were mixed in a 50-mL round-bottom flask. TCEP·HCl (0.100 g, 3.49×10^{-4} mol) was added to the reaction mixture, which was stirred at room temperature for 3.5 h under N_2. The reaction mixture was poured into 50 mL of 0.2 M $NaHCO_3$ solution. The product was extracted with $CHCl_3$ (4 times). The combined organic layers were dried over Na_2SO_4. After the inorganic solid was filtered off, the solvent was evaporated to obtain the product as transparent oil. Yield = 0.168 g (~68% based on 0.247 g of TA-PEG600-NTA(OMe)(OEt)$_2$). ^{1}H NMR (400 MHz, $CDCl_3$): δ 6.65 (br s, 1H, N*H*), 6.43 (br s, 1H, N*H*), 6.28 (br s, 1H, N*H*), 4.1–4.2 (m, 4H, –OC*H*$_2$CH$_3$), 3.59–3.76 (m), 3.51–3.59 (m, 4H), 3.38–3.51 (m, 5H), 3.16–3.31 (m, 2H), 2.86–2.99 (m, 1H),

2.60–2.81 (m, 2H), 2.51 (s, 4H), 2.20 (t, 2H, *J* = 9.8 Hz, –C*H*$_2$CO–), 1.84–1.97 (m, 1H), 1.4–1.8 (m, 13H), 1.36 (t, 1H, *J* = 10.7 Hz, –S*H*), 1.31 (d, 1H, *J* = 10.1 Hz, –S*H*), 1.26 (t, 6H, *J* = 9.5 Hz, –OCH$_2$C*H*$_3$).

Ligand Exchange onto Quantum Dots. The hydrophobic QDs were made hydrophilic by exchanging the native ligands with the customized ligands containing dihydrolipoic acid (DHLA) using either a biphasic mixture method for CL4 or a premetallation method for DHLA-PEG$_{750}$-OMe and DHLA-PEG$_{600}$-NTA(OMe)(OEt)$_2$ as previously reported. The terminal methyl and ethyl ester groups of DHLA-PEG$_{600}$-NTA(OMe)(OEt)$_2$ were hydrolyzed in 0.1 M NaOH solution after the ligand exchange to expose carboxyl groups.

2. Supporting Information Tables

Table S1. Fluorescence microscopy imaging filter sets used in this study.

Imaging Filter Set	Excitation Filter	Dichroic Filter	Emission Filter
DIC [a]	Open	400 nm	Open
QD [b]	410 ± 30 nm	510 nm longpass	535 ± 50 nm
MCherry[b]	576 ± 23 nm	595 nm longpass	620 ± 40 nm
QD-mCherry FRET[b]	410 ± 30 nm	595 nm longpass	620 ± 40 nm

[a] DIC (differential interference contrast) imaging was performed using a white light illumination source, a polarizer and a Wollaston prism. Images were false-colored grey for visual clarity; [b] UV excitation was performed using a metal halide short-arc lamp.

3. Supporting Information Figures

```
ggtaccgccatggcgctgctgccatttcaactgttagctgttctctttcctggtggtaac
 G  T  A  M  A  L  L  P  F  Q  L  L  A  V  L  F  P  G  G  N
agtgaacatgccttccagggccgacctcctttcatgttatccagacctcgtcctttacc
 S  E  H  A  F  Q  G  P  T  S  F  H  V  I  Q  T  S  S  F  T
aatagtacctgggcacaaactcaaggctcaggctggttggatgatttgcagattcatggc
 N  S  T  W  A  Q  T  Q  G  S  G  W  L  D  D  L  Q  I  H  G
tgggatagcgactcaggcactgccatattcctgaagccttggtctaaaggtaactttagt
 W  D  S  D  S  G  T  A  I  F  L  K  P  W  S  K  G  N  F  S
gataaggaggttgctgagttagaggagatattccgagtctacatctttggattcgctcga
 D  K  E  V  A  E  L  E  E  I  F  R  V  Y  I  F  G  F  A  R
gaagtacaagactttgccggtgatttccagatgaaataccccttgagatccagggcata
 E  V  Q  D  F  A  G  D  F  Q  M  K  Y  P  F  E  I  Q  G  I
gcaggctgtgagctacattctggaggtgccatagtaagcttcctgaggggagctctagga
 A  G  C  E  L  H  S  G  G  A  I  V  S  F  L  R  G  A  L  G
ggattggatttcctgagtgtcaagaatgcttcatgtgtgccttccccagaaggtggcagc
 G  L  D  F  L  S  V  K  N  A  S  C  V  P  S  P  E  G  G  S
agggcacagaaattctgtgcactaatcatacaatatcaaggtatcatggaaactgtgaga
 R  A  Q  K  F  C  A  L  I  I  Q  Y  Q  G  I  M  E  T  V  R
attctcctctatgaaacctgcccccgatatctcttgggcgtcctcaatgcaggaaaagca
 I  L  L  Y  E  T  C  P  R  Y  L  L  G  V  L  N  A  G  K  A
gatctgcaaagacaagtgaagcctgaggcctggctgtccagtggccccagtcctggacct
 D  L  Q  R  Q  V  K  P  E  A  W  L  S  S  G  P  S  P  G  P
ggccgtctgcagcttgtgtgccatgtctcaggattctacccaaagcccgtgtgggtgatg
 G  R  L  Q  L  V  C  H  V  S  G  F  Y  P  K  P  V  W  V  M
tggatgcggggtgagcaggagcagcagggcactcagctaggggacatcctgcccaatgct
 W  M  R  G  E  Q  E  Q  Q  G  T  Q  L  G  D  I  L  P  N  A
aactggacatggtatctccgagcaaccctggatgtggcagatggggaggcggctggcctg
 N  W  T  W  Y  L  R  A  T  L  D  V  A  D  G  E  A  A  G  L
tcctgtcgggtgaagcacagcagtttagagggccaggacatcatcctctactggagaaac
 S  C  R  V  K  H  S  S  L  E  G  Q  D  I  I  L  Y  W  R  N
cccacctccattggctcaattgttttggcaataatagtgccttccttgctcctttttgcta
 P  T  S  I  G  S  I  V  L  A  I  I  V  P  S  L  L  L  L  L
tgccttgcattatggtatatgaggcgccggtcatatcagaatatcccaggggcggatcc
 C  L  A  L  W  Y  M  R  R  R  S  Y  Q  N  I  P     G  G  G  S
atggtgagcaagggcgaggaggataacatggccatcatcaaggagttcatgcgcttcaag
 M  V  S  K  G  E  E  D  N  M  A  I  I  K  E  F  M  R  F  K
gtgcacatggagggctccgtgaacggccacgagttcgagatcgagggcgagggcgagggc
 V  H  M  E  G  S  V  N  G  H  E  F  E  I  E  G  E  G  E  G
cgcccctacgagggcacccagaccgccaagctgaaggtgaccaagggtggccccctgccc
 R  P  Y  E  G  T  Q  T  A  K  L  K  V  T  K  G  G  P  L  P
ttcgcctgggacatcctgtcccctcagttcatgtacggctccaaggcctacgtgaagcac
 F  A  W  D  I  L  S  P  Q  F  M  Y  G  S  K  A  Y  V  K  H
cccgccgacatccccgactacttgaagctgtccttccccgagggcttcaagtgggagcgc
 P  A  D  I  P  D  Y  L  K  L  S  F  P  E  G  F  K  W  E  R
gtgatgaacttcgaggacggcggcgtggtgaccgtgacccaggactcctccctgcaggac
V   M  N  F  E  D  G  G  V  V  T  V  T  Q  D  S  S  L  Q  D
ggcgagttcatctacaaggtgaagctgcgcggcaccaacttcccctccgacggccccgta
 G  E  F  I  Y  K  V  K  L  R  G  T  N  F  P  S  D  G  P  V
atgcagaagaagaccatgggctgggaggcctcctccgagcggatgtaccccgaggacggc
 M  Q  K  K  T  M  G  W  E  A  S  S  E  R  M  Y  P  E  D  G
aacatcaagttggacatcacctcccacaacgaggactacaccatcgtggaacagtacgaa
 N  I  K  L  D  I  T  S  H  N  E  D  Y  T  I  V  E  Q  Y  E
gaggtcaagaccacctacaaggccaagaagcccgtgcagctgcccggcgcctacaacgtc
 E  V  K  T  T  Y  K  A  K  K  P  V  Q  L  P  G  A  Y  N  V
gccctgaagggcgagatcaagcagaggctgaagctgaaggacggcggccactacgacgct
 A  L  K  G  E  I  K  Q  R  L  K  L  K  D  G  G  H  Y  D  A
gaggtcaagaccacctacaaggccaagaagcccgtgcagctgcccggcgcctacaacgtc
 E  V  K  T  T  Y  K  A  K  K  P  V  Q  L  P  G  A  Y  N  V
aacatcaagttggacatcacctcccacaacgaggactacaccatcgtggaacagtacgaa
 N  I  K  L  D  I  T  S  H  N  E  D  Y  T  I  V  E  Q  Y  E
cgcgccgagggccgccactccaccggcggcatggacgagctgtacaaggcggccgc
 R  A  E  G  R  H  S  T  G  G  M  D  E  L  Y  K  A  A …myc tag…his(6)tag
```

Figure S1. Nucleotide and translated amino acid sequence of the CD1b-mCherry construct. Shown are the nucleotide and translated amino acid sequence of the membrane-localized CD1b-mCherry construct used in these studies. The various domains are color-coded as indicated. The *Kpn*I and *Not*I restriction sites used to clone the as-synthesized gene sequence into the pcDNA3.1-myc-his (A) vector are underlined. The domains are color-coded as follows: 1-yellow: Signal sequence to guide the insertion of the protein into the secretory pathway is (cleaved in the mature protein); 2-red: Extracellular domain of CD1b; 3-magenta: Transmembrane-spanning domain of CD1b; 4-blue: Cytosolic domain of CD1b; 5-green: Flexible linker/spacer; 6-grey: mCherry. The mature protein contains a C-terminal His_6 domain for assembly to QDs (**bold**).

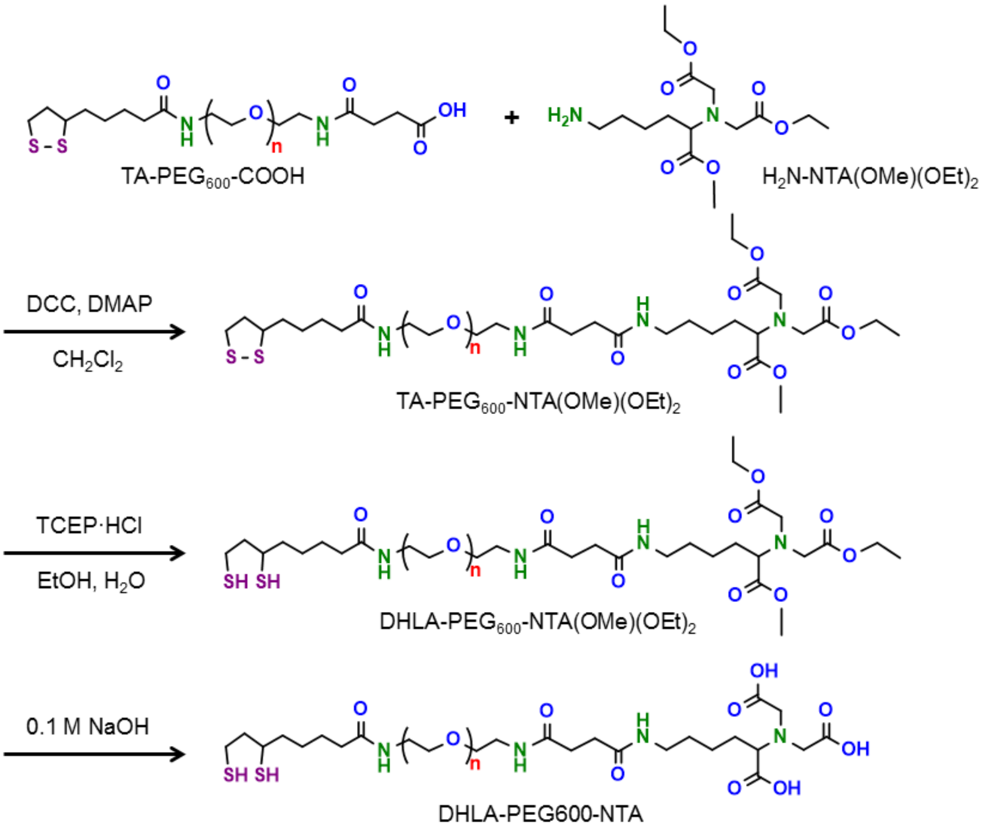

Figure S2. Synthetic scheme for ligand DHLA-PEG600-NTA.

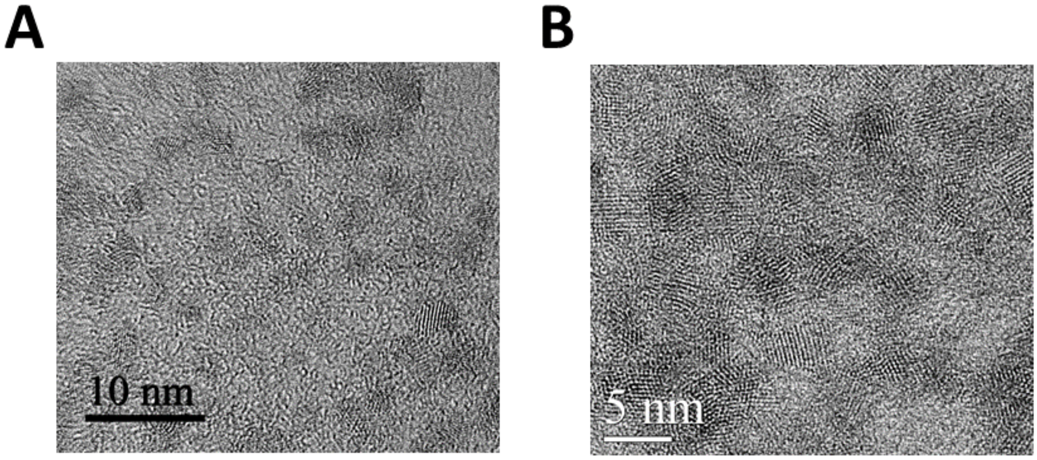

Figure S3. Representative transmission electron microscopy analysis of QDs used in this study. (**A**) QDs capped with CL4 and DHLA-PEG600-NTA had average diameter of 4.6 ± 0.4 nm; (**B**) QDs capped with DHLA-PEG750-OMe had average diameter of 4.7 ± 0.4 nm.

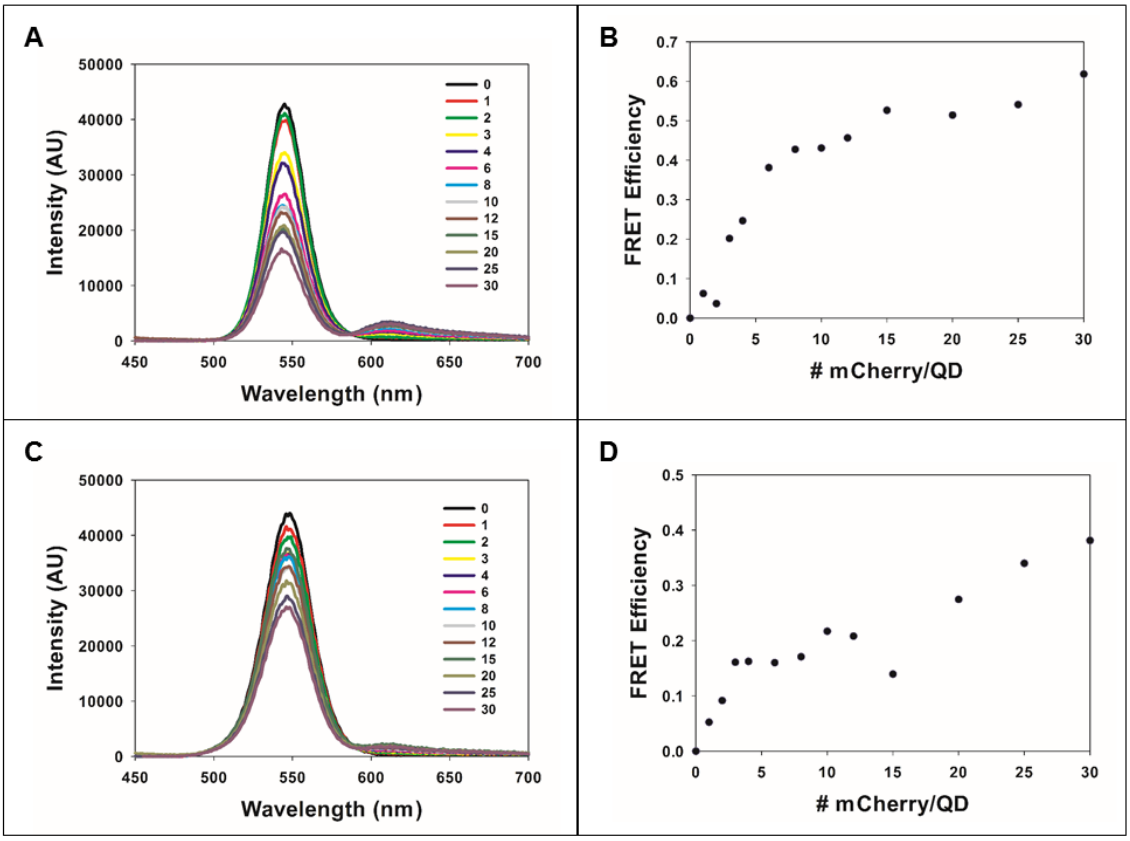

Figure S4. Förster resonance energy transfer (FRET) analysis for nitrilotriacetic acid (PEG_{600}-NTA) and methoxy (PEG_{750}-OMe) capped QDs. (**A**) Emission spectra of PEG_{600}-NTA capped 550 nm QDs showing sensitization of QD donor emission with increasing ratio of mCherry acceptor; (**B**) Plot of QD-mCherry FRET efficiency for PEG_{600}-NTA-capped QDs; (**C**) Emission spectra of PEG_{750}-OMe capped 550 nm QDs showing sensitization of QD donor emission with increasing ratio of mCherry acceptor. Data has been corrected for direct excitation of mCherry; (**D**) Plot of QD-mCherry FRET efficiency for PEG_{750}-OMe-capped QDs.

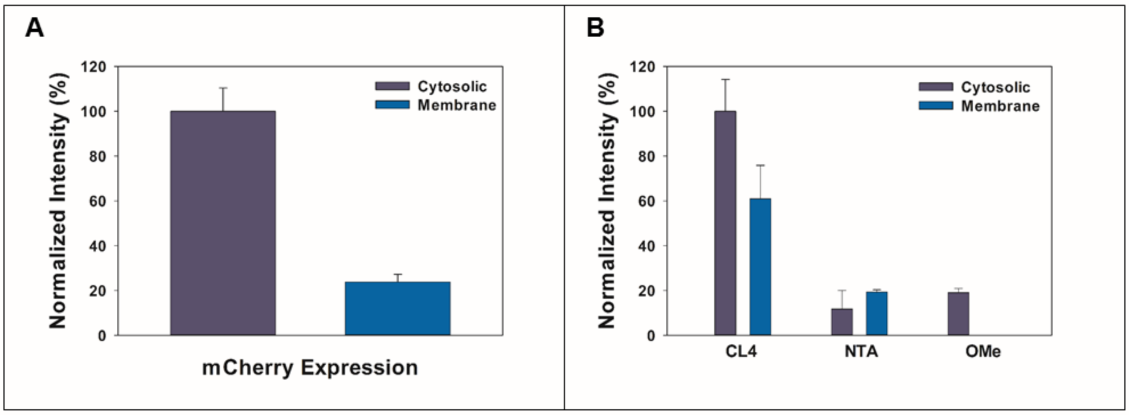

Figure S5. Relative quantification of cytosolic *vs.* membrane-localized mCherry expression and ligand-dependent FRET intensity. (**A**) Normalized fluorescence intensity of mCherry expression level in the cysotol *vs.* localized to the plasma membrane; (**B**) Normalized comparison of FRET intensities of mCherry expressed in the cytosol *vs.* at the plasma membrane as a function of QD-capping ligand. The FRET intensities for the PEG_{60}0-NTA and PEG_{750}-OMe ligands are normalized to the intensity of CL4. No FRET intensity could be measured for OMe-capped QDs at the plasma membrane. The data are expressed as the average FRET intensity ± standard error of the mean.

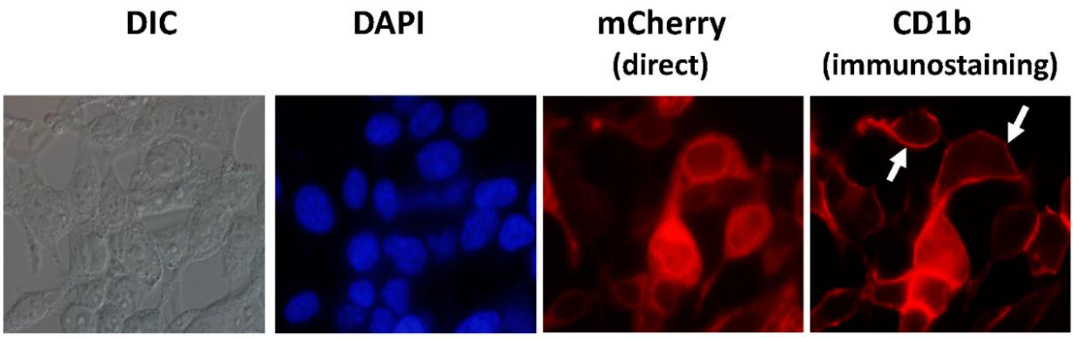

Figure S6. Confirmation/characterization of expression of mCherry as membrane fusion to CD1b. Shown is the expression pattern of mCherry when expressed on the inner (cytofacial) leaflet of the plasma membrane as a C-terminal fusion to the extracellular and transmembrane domains of CD1b in HEK 293T/17 cells. See Figure S1 for nucleotide and amino acid sequence. mCherry fluorescence was visualized via direct excitation using the imaging settings in Table S1. The extracellular domain of CD1b was probed/visualized using an anti-human CD1b antibody conjugated to 660 nm-emitting nanocrystals (eBioscience/Affymetrix) after the cells were fixed with 4% paraformaldehyde. The CD1b staining clearly shows the localization of the CD1b-mCherry fusion to the plasma membrane (arrows).

References

1. Dwyer, C.L.; Díaz, S.A.; Walper, S.A.; Samanta, A.; Susumu, K.; Oh, E.; Buckhout-White, S.; Medintz, I.L. Chemoenzymatic sensitization of DNA photonic wires mediated through quantum dot energy transfer relays. *Chem. Mater.* **2015**, *27*, 6490–6494.
2. Susumu, K.; Uyeda, H.T.; Medintz, I.L.; Pons, T.; Delehanty, J.B.; Mattoussi, H. Enhancing the stability of quantum dots via compact multifunctional ligands. *J. Am. Chem. Soc.* **2007**, *129*, 13987–13996.
3. Susumu, K.; Mei, B.C.; Mattoussi, H. Multifunctional ligands based on dihydrolipoic acid and polyethylene glycol to promote biocompatibility of quantum dots. *Nat. Protoc.* **2009**, *4*, 424–436.
